# Generational Differences in Food Consumption among Chinese Adults of Different Ages

**DOI:** 10.3390/nu15204451

**Published:** 2023-10-20

**Authors:** Lijie Guo, Feifei Huang, Mengran Liu, Yueyang Zhang, Jiguo Zhang, Bing Zhang, Huijun Wang

**Affiliations:** 1 National Institute for Nutrition and Health, Chinese Center for Disease Control and Prevention, Beijing 100050, China; guolijie0002021@163.com (L.G.);; 2 Department of Education and Training, Chinese Center for Disease Control and Prevention, Beijing 102206, China; 3 Department of Health Services, Policy & Practice, School of Public Health, Brown University, Providence, RI 02903, USA; 4 Key Laboratory of Trace Element Nutrition of Health Commission of China, Beijing 100050, China

**Keywords:** dietary, food consumption, generational differences, China Health and Nutrition Survey

## Abstract

Limited knowledge currently exists regarding the dynamics of generational shifts in food consumption among adult residents in China. This study aimed to investigate the generational differences in dietary status among different generations of Chinese adult residents aged 20 years and older. Survey participants from four waves (1991, 2000, 2009, and 2018) of the China Health and Nutrition Survey (CHNS) cohort were included in the study (N = 40,704), providing three-day 24 h dietary data. Participants were categorized into six age groups (20–29, 30–39, 40–49, 50–59, 60–69, and ≥70 years old), each corresponding to specific generations (Gen 10, 20, 30, 40, 50, 60, 70, 80, and 90) based on their age at the time of the survey. This study examined generational differences in the intake of cereals, animal-based foods, vegetables, fruits, dairy, energy, and the contribution of macronutrients to energy using chi-square tests and Kruskal–Wallis tests. All analyses were stratified by gender. Our findings revealed that younger generations exhibited lower daily intake of cereals, vegetables, energy, and contribution of carbohydrates to energy, compared to their older counterparts, regardless of gender. Conversely, regardless of male or female, younger generations showed higher daily consumption of animal-based foods, average fruit and dairy intake, fruit and dairy consumption rates, as well as contributions of protein and fat to energy, compared to older generations. The magnitude of generational differences in food consumption varied by age and gender. In addition, cereal and vegetable intake, energy intake, and contribution of carbohydrates to energy declined with age across all generations, while average dairy intake, fruit and dairy consumption rates, and the contribution of fat to energy tended to increase, regardless of gender. In conclusion, generational differences in food consumption were evident among different generations of Chinese adult residents, characterized by an increase in animal-based food intake and the contribution of fat to energy among generations. Attention should be directed towards addressing the eating behavior of younger generations.

## 1. Introduction

The food and nutrition of a population are closely related to the economic and social development of a country or region. Food consumption is determined by individual and contextual factors, including the food environment [1]. Acting as the arena where food purchases occur, the food environment is influenced and constrained by market dynamics, encompassing community, consumer, and organizational food environment [2]. Previous research has evidenced that the quality of the food environment is independently related to food consumption [1,3]. In particular, the presence of “food deserts” can lead to social disparities in diet and diet-related health outcomes, such as cardiovascular disease and obesity [3]. China’s burgeoning food consumption landscape and shifts in the nutritional status of its population are intertwined with various food environment factors. These factors included income levels, food prices, regional development, food production capacity, market expansion, and the level of advancement in the catering industry [4]. It’s worth noting that the impact of China’s food environment may vary by population groups. Geographical disparities in community food environments within China exert varying degrees of influence on the dietary intake, dietary behaviors, and nutritional status of the adult population [5,6]. Research has underscored the rapid expansion of China’s fast-food industry and the phenomenal growth of the online food sector over the past two decades, creating substantial opportunities for enhanced food accessibility and availability [7]. However, this expansion has also posed inevitable challenges to the public health system and the overall food environment [8].

Generational differences in diet arise from the shared exposure of individuals within the same generation to similar historical and social contexts from birth. These collective experiences may influence their lifestyle and dietary behaviors throughout their lifespans, leading to significantly divergent dietary habits among different generations [9]. For instance, a study examining generational differences in energy, macronutrient, and food group intakes among older adults aged 55 years and above in the United States from 1977 to 2010 found that energy intake declined with age in older generations. However, this decline in energy consumption was absent in younger generations from age 55 onwards [9]. Another study exploring the impact of generational status on the dietary behaviors of Asian Americans indicated that second and third generations reported higher intake of red meat and processed meat compared to the first generation (+0.044 times/day, +0.029 times/day; +0.061 times/day) [10]. In New Zealand, the mean food scores of Pasifika residents exhibited statistically significant differences in dietary patterns between older and younger generations. Older generations displayed greater diversity in their food consumption, characterized by predominantly “healthy diets.” In contrast, younger generations were more likely to consume “processed diets” [11]. Generational differences in nutritional status also manifested in two generations of Chinese residents born in the 1960s and 1980s. The contribution of fat to energy intake and cholesterol intake exceeding the Chinese dietary reference intakes (Chinese DRIs) were all significantly higher among residents born in the 1980s than those born in the 1960s. Conversely, the energy intake, salt intake, and the contribution of carbohydrates to energy intake exceeding the Chinese DRIs of residents born in the 1980s were all significantly lower than in residents born in the 1960s [12]. Despite numerous domestic and international studies exploring current dietary patterns and trends in food intake, there remains a dearth of research investigating generational differences in food consumption among different generations.

The objective of this study was to describe the food consumption, energy intake, and the contribution of macronutrients to energy among Chinese adult residents aged 20 years and older across different generations, while also exploring generational differences.

## 2. Materials and Methods

### 2.1. Study Design and Population

The China Health and Nutrition Survey (CHNS) is an ongoing, longitudinal, and prospective cohort study in China. The first round of surveys began in 1989 and by 2018, a total of 11 rounds of surveys were conducted, covering 15 provinces (autonomous regions and municipalities) including Beijing, Liaoning, Heilongjiang, Shanghai, Jiangsu, Zhejiang, Shandong, Henan, Hubei, Hunan, Guangxi, Chongqing, Guizhou, Yunnan and Shaanxi. Selecting two cities and four counties in each province (autonomous region or municipality directly under the Central Government), two neighborhood committees and two suburban villages were randomly selected at each selected city point; each prefecture selects one neighborhood council and three natural villages, where the prefecture government is located; each survey site (neighborhood committee/village) randomly selects 20 households, and all family members in the survey household are the survey subjects. The first wave of the CHNS survey utilized a stratified multistage cluster random sampling method. Subsequent waves of the survey were conducted to follow up with the first sample cohort. If additional sites are added, the sample is still sampled using the multistage stratified cluster random sampling method. The survey collected demographic information and dietary data from 15 provinces. Specific sampling methods, survey programs, and quality control measures are described in the references [13,14]. The study was conducted in accordance with the Declaration of Helsinki, and approved by the Institutional Review Board of the University of North Carolina at Chapel Hill (No. 07-1963), the Institutional Review Committee of the National Institute for Nutrition and Health, and the Chinese Center for Disease Control and Prevention approved the survey protocols, instruments, and procedures for obtaining informed consent (No. 201524). Informed consent forms were signed by all respondents and their guardians prior to participation in the survey.

This study leveraged data from four waves of the CHNS conducted in 1991, 2000, 2009, and 2018. A total of 41,125 subjects aged 20 years and older, who provided both dietary and demographic information data—excluding pregnant or lactating women and individuals with cancer—were included at baseline. After applying these criteria, 421 participants with implausible energy intakes were further excluded (men: <800 kcal/day or >6000 kcal/day; women: <600 kcal/day or >4000 kcal/day) [15]. Ultimately, a final cohort of 40,704 participants was included for analysis in this study. The number of participants was 7749 (19.1%) in 1991, 9324 (22.9%) in 2000, 9370 (23.0%) in 2009, and 14,261 (35.0%) in 2018.

The study population was divided into six age groups based on their age at the time of the survey: 20–29 years, 30–39 years, 40–49 years, 50–59 years, 60–69 years, and 70 years and older. Each age group corresponded to nine distinct generational cohorts, denoted as generation 10, 20, 30, 40, 50, 60, 70, 80, and 90, across the four survey waves (Table 1). The dietary intakes of individuals born in different generations but of similar ages (indicated in the charts by age group means: 25, 35, 45, 55, 65, and 75 years old) were compared to analyze the generational differences.

### 2.2. Dietary Assessment and Other Measurements

Dietary data were collected through a series of consecutive three-day 24 h dietary recalls (two weekdays and one weekend day), alongside household seasoning consumption data recorded using the household food weighing method. This approach aimed to capture the average daily intake of various foods per individual. Furthermore, the average daily energy intake, the contribution of carbohydrates to energy, the contribution of fat to energy, and the contribution of protein to energy per individual were calculated according to the Chinese Food Composition Table [16]. The food groups were classified into five categories, aligning with the definitions of food classification in the Chinese Dietary Guidelines [17], including cereal, animal-based food, vegetables, fruit, and dairy.

In addition to the dietary data, we gathered baseline information about the participants through an interview questionnaire. This questionnaire aimed to collect primary demographic data, encompassing age, gender, education level (elementary school and below, middle school, high school and above), place of residence, work situation, and individual annual income (grouped by tertiles of the data distribution).

### 2.3. Statistical Analysis

The categorical variables were presented using count (n) and percentage (%), while continuous variables were described using median (M), Interquartile Range (IQR), mean, and standard deviation (SD). To compare generational differences across the different generations, the χ^2^ test was employed for categorical variables, and the Kruskal–Wallis test was utilized for continuous variables.

Throughout this study, all analyses were stratified by gender. Data cleaning and analysis were performed using SAS version 9.4 (SAS Institute, Inc., Cary, NC, USA) statistical software. A significant level of *p* < 0.05 was considered statistically significant. Additionally, the Bonferroni method was applied to correct the test levels when comparing two groups, with *p* < 0.0083 considered statistically significant.

## 3. Results

### 3.1. Population Characteristics

The basic characteristics of the study population are presented in Table 2. Over the years 1991 to 2018, the average age of participants showed a steady increase (males: 42.7, 45.7, 50.4, 54.4 years old; females 43.3, 47.2, 51.4, 54.4 years old). Across all four survey waves, the percentage of participants aged 70 years and older remained consistently the lowest, while a higher percentage of participants resided in rural areas compared to urban areas. The highest percentage of participants in 2018 was found in both men and women with an education level of senior high school and above.

### 3.2. Generational Differences in Food Consumption

**Figure 1:**
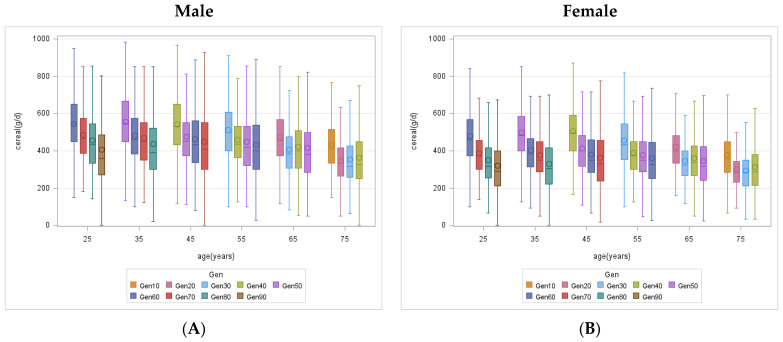

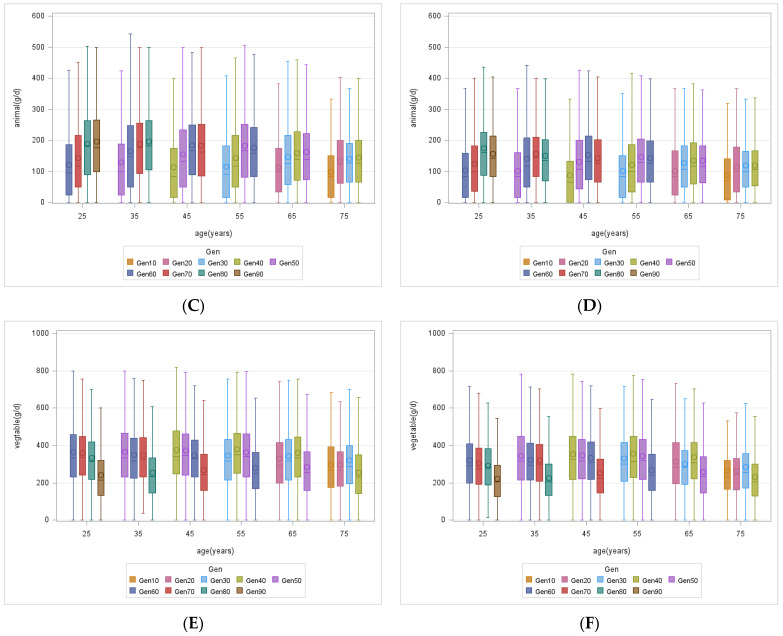
Generational differences in food consumption among Chinese residents aged 20 and older. The analysis was stratified by gender: (**A**,**B**) cereal intake; (**C**,**D**) animal-based food intake; and (**E**,**F**) vegetable intake. Gen, generation. The origin in the figure represents the average food consumption.

Figure 1 illustrates the progression of food intake in China among different generations of residents. Notably, significant generational differences in daily cereal intake, animal-based food intake, and vegetable intake were observed for both males and females in all age groups (*p* < 0.0001). Among males and females aged 20–59 years, the younger generation exhibited lower daily cereal intake compared to older generations (median cereal intake for males aged 40–49 years: Gen 40 (533.3 g/d), Gen 50 (450.0 g/d), Gen 60 (433.3 g/d), and Gen 70 (409.3 g/d); and the corresponding median cereal intake for females aged 40–49 years: Gen 40 (496.7 g/d), Gen 50 (400.0 g/d), Gen 60 (350.0 g/d), Gen 70 (332.1 g/d) (Figure 1A,B). Except for the youngest generation, individuals aged 20–59 years from younger generations had higher median animal-based food intake than the older generations (median animal food intake for males aged 40–49 years: Gen 40 (84.6 g/d), Gen 50 (133.3 g/d), Gen 60 (166.7 g/d); and for females aged 40–49 years: Gen 40 (65.0 g/d), Gen 50 (108.3 g/d), Gen 60 (133.3 g/d)) (Figure 1C,D). Furthermore, generational differences in vegetable intake existed between the youngest two generations and were significantly lower in the younger than in the older generations (median vegetable intake for males aged 40–49 years: Gen 60 (330.0 g/d), Gen 70 (243.3 g/d); and for females aged 40–49 years: Gen 60 (310.0 g/d), Gen 70 (225.5 g/d)) (Figure 1E,F). The generational trends in the intake of various food groups were similar for males and females, but the extent of generational changes in the intake of some food groups diverged. The generational increment in animal-based food intake was higher for males than females. The generational reduction in vegetable intake was larger for males than for females.

Across all generations, cereal and vegetable intake exhibited a tendency to decline with age in both men and women. In contrast, animal-based food intake experienced an initial increase followed by a subsequent decline and converged after the age of 70 years.

### 3.3. Generational Differences in Fruit and Dairy Consumption

**Figure 2:**
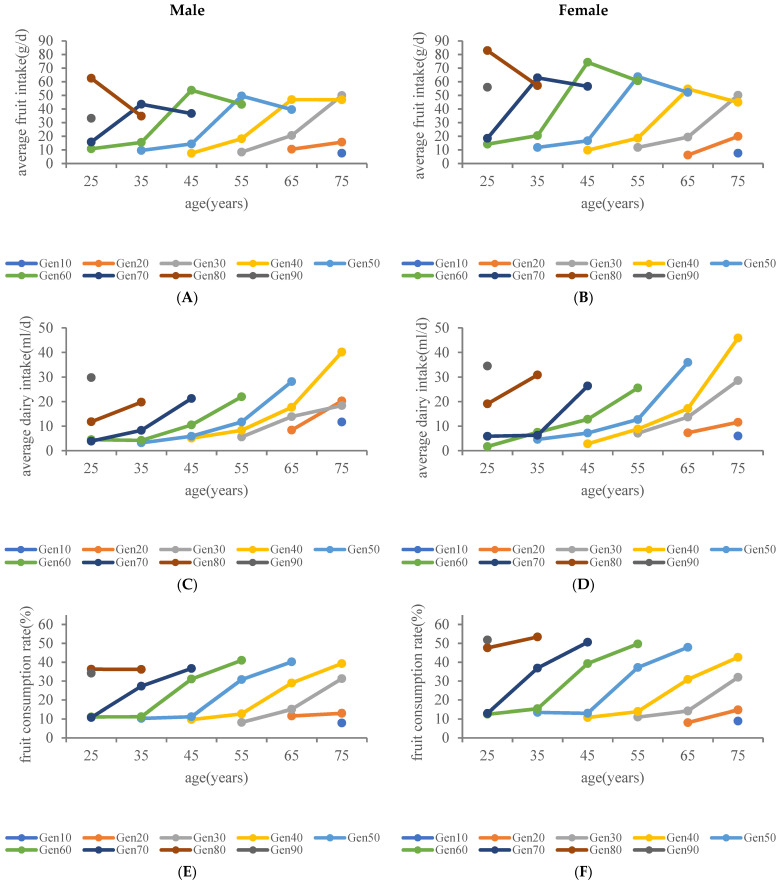

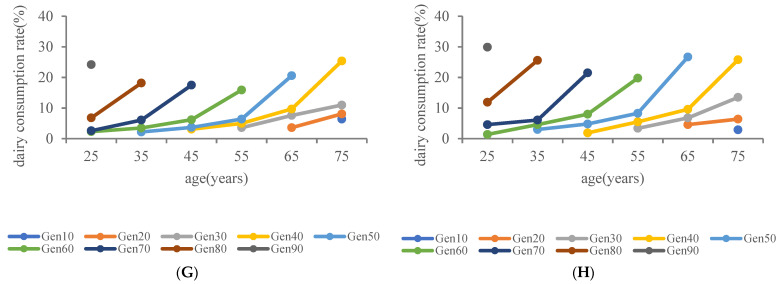
Generational differences in fruit and dairy consumption among Chinese residents aged 20 and older. The analysis was stratified by gender: (**A**,**B**) average fruit intake; (**C**,**D**) average dairy intake; (**E**,**F**) fruit consumption rate; (**G**,**H**) dairy consumption rate. Gen, generation.

Figure 2 showcases the progression of fruit and dairy intake in China among different generations of residents. There were significant generational differences in daily average fruit intake and dairy intake for both males and females across all age groups (*p* < 0.0001). Average fruit intake was higher in the third generation than in the second generation in both men and women of all age groups (*p* < 0.0083) (Mean fruit intake of men aged 40–49 years: Gen 50 (14.4 g/d), Gen 60 (53.8 g/d); and for females aged 40–49 years: Gen 50 (16.7 g/d), Gen 60 (74.3 g/d)) (Figure 2A,B). Additionally, in all age groups for both genders, the youngest generation displayed a higher average dairy intake compared to the previous generation (*p* < 0.0083) (mean dairy intake for males aged 40–49 years: Gen 60 (10.5 mL/d), Gen 70 (21.3 mL/d); and for females aged 40–49 years: Gen 60 (12.8 mL/d), Gen 70 (26.4 mL/d)) (Figure 2C,D). The generational increment in mean fruit intake was much greater in women than in men.

Furthermore, except for the oldest generation, the fruit consumption rate of younger generations surpassed that of their predecessors (the fruit consumption rate of men aged 40–49 years: Gen 50 (11.2%), Gen 60 (31.1%), Gen 70 (36.7%); for females aged 40–49 years: Gen 50 (13.0%), Gen 60 (39.3%), Gen 70 (50.6%)) (Figure 2E,F). Similarly, the dairy consumption rate in the youngest generation exceeded that of the previous generation (the dairy consumption rate of men aged 40–49 years: Gen 60 (6.2%), Gen 70 (17.5%); for females aged 40–49 years: Gen 60 (8.0%), Gen 70 (21.5%) (Figure 2G,H). The generational growth in the fruit consumption rate was greater for females than for males.

Across all generations, a trend of increasing average dairy intake, fruit consumption rate, and dairy consumption rate with age was observed in both males and females.

### 3.4. Generational Differences in Energy Intake and the Contribution of Macronutrients to Energy

**Figure 3:**
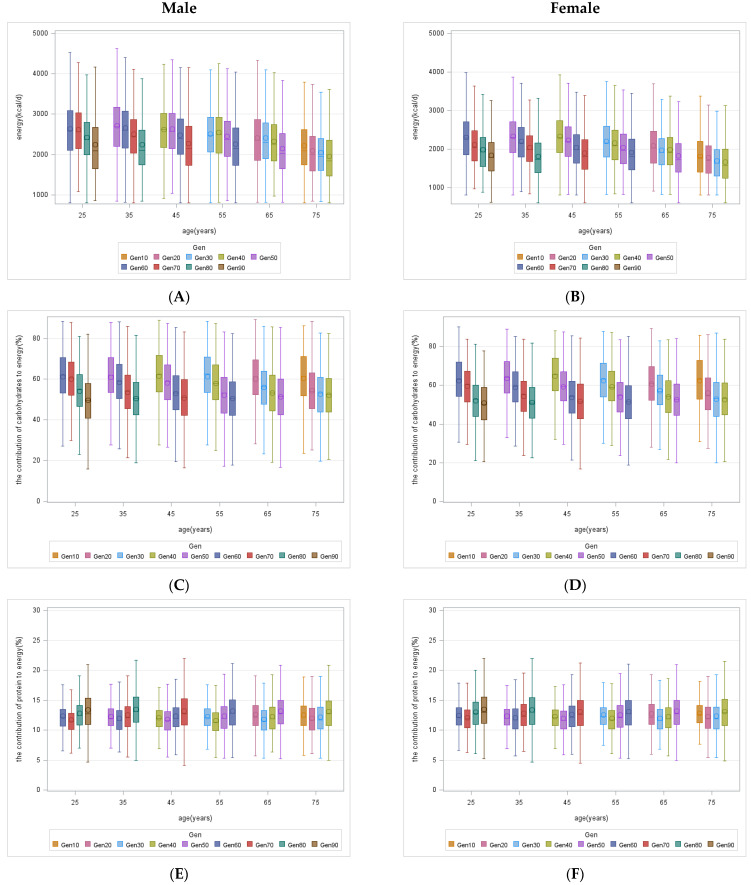

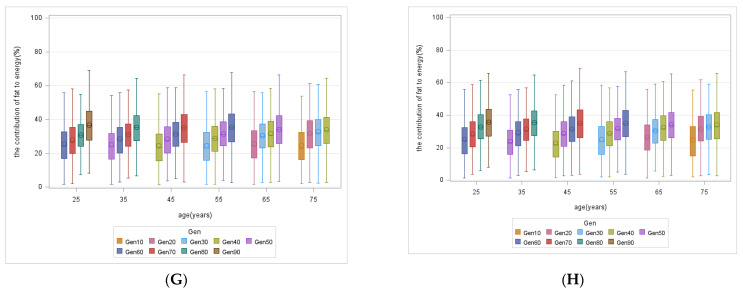
Generational differences in energy and macronutrients among Chinese residents aged 20 years and older. The analysis was stratified by gender: (**A**,**B**) energy; (**C**,**D**) the contribution of carbohydrates to energy; (**E**,**F**) the contribution of protein to energy; (**G**,**H**) the contribution of fat to energy. The origin in the figure represents the average food consumption.

Figure 3 demonstrates the progression of energy intake and the contribution of macronutrients to energy among different generations of Chinese residents. Significant generational differences (*p* < 0.0001) were observed in daily energy intake, the contribution of carbohydrates to energy, the contribution of protein to energy, and the contribution of fat to energy for men and women in all age groups (*p* < 0.0001). With the exception of the oldest generation, younger generations of males and females aged 20–59 years exhibited lower daily energy intakes compared to their older counterparts (median energy intake for males aged 40–49 years: Gen 50 (2573.3 kcal/d), Gen 60 (2392.0 kcal/d), Gen 70 (2148.7 kcal/d); median energy intake for females aged 40–49 years: Gen 50 (2197.5 kcal/d), Gen 60 (1979.8 kcal/d), Gen 70 (1812.6 kcal/d)) (Figure 3A,B). Additionally, the younger generations aged 30–69 years displayed a lower contribution of carbohydrates to energy than the older generations (median contribution of carbohydrates to energy for males aged 40–49 years: Gen 40 (62.2%), Gen 50 (59.1%), Gen 60 (53.4%), Gen 70 (51.1%); median contribution of carbohydrates to energy for females aged 40–49 years: Gen 40 (65.6%), Gen 50 (59.5%), Gen 60 (54.3%), Gen 70 (51.6%)) (Figure 3C,D). Conversely, younger generations aged 30–59 years exhibited a higher contribution of protein to energy than the older generations (median contribution of protein to energy in males aged 40–49 years: Gen 40 (11.9%), Gen 50 (11.6%), Gen 60 (12.0%), Gen 70 (12.7%); median contribution of protein to energy in females aged 40–49 years: Gen 40 (12.0%), Gen 50 (11.6%), Gen 60 (12.1%), Gen 70 (12.6%)) (Figure 3E,F). Furthermore, the median contribution of fat to energy was higher in younger than older generations aged 20–69 years (median contribution of fat to energy in men aged 40–49 years: Gen 40 (23.5%), Gen 50 (27.8%), Gen 60 (31.4%), Gen 70 (35.0%); median contribution of fat to energy in women aged 40–49 years: Gen 40 (21.7%), Gen 50 (28.0%), Gen 60 (31.4%), Gen 70 (34.7%)) (Figure 3G,H). The generational increase in energy intake and contribution of fat to energy was higher in males than in females.

Across all generations, energy intake and the contribution of carbohydrates to energy tended to decrease with age in both males and females, while the contribution of fat to energy tended to increase, with all three converging after age 70. In contrast, the contribution of protein to energy displayed an initial decline followed by an increase.

## 4. Discussion

This study revealed generational differences in food consumption, energy intake, and the contribution of macronutrients to energy among Chinese residents aged 20 years and older. Younger generations exhibited lower daily cereal and vegetable intake, as well as reduced energy intake and contribution of carbohydrates to energy when compared to older generations (*p* < 0.0001). On the contrary, daily intake of animal-based foods, average fruit and dairy intakes, fruit and dairy consumption rates, and contributions of protein and fat to energy were higher in the younger generation than in the older generation. These findings are consistent with prior studies conducted in China, indicating that the energy intake and contribution of carbohydrates to energy of Gen 80 were significantly lower than that of Gen 60, while the contribution of fat to energy was significantly higher than that of Gen 60 [12].

In addition, our study found that cereal and vegetable intakes, energy intake, and contribution of carbohydrates to energy tended to decrease with age across all generations. Conversely, average dairy intake, fruit and dairy consumption rates, and the contribution of fat to energy tended to increase across all generations. Animal-based food intake experienced an initial rise followed by a subsequent decline that converged after age 70, while the contribution of protein to energy demonstrated an initial decline and subsequent increase. These trends are consistent with previous research findings. For instance, dietary energy intake and the contribution of carbohydrates to energy in Chinese adults aged 18–35 years displayed a decreasing trend, while the contribution of fat to energy gradually increased between 1989 and 2018 [18,19]. Furthermore, vegetable intake exhibited a downward trend, whereas fruit and dairy intake showed an upward trend [20].

Several potential factors contributed to the generational differences in food consumption among different generations of Chinese residents. Economic and social development has led to increased household incomes and greater purchasing power among recent generations. In addition, the modern commodity system has facilitated easier access to food, providing residents with more convenience and freedom in their food choices [21,22]. Moreover, the influence of information media played a pivotal role in shaping the dietary preferences of younger generations. Several studies underscored that Western dietary patterns gained prominence in China due to media exposure, leading to the adoption of habits commonly observed in Western countries such as Europe and the United States. These patterns, characterized by high-energy-density, high-fat, and low-dietary-fiber foods such as processed meats and dairy products, have become increasingly popular among Chinese children and adolescents. In tandem, the pervasive reach of the Internet has further amplified these trends, transcending temporal and geographical constraints and fostering the growth of the catering industry platform economy [22]. Concurrently, the contemporary fast-paced work environment and lifestyle have driven an increase in dining outside the home and the frequency of consuming fast food among recent generations in China. The propensity to dine out usually resulted in a heightened intake of energy, fat, protein, sugar, and alcohol [23].

Studies conducted in other countries have also indicated that younger generations tend to have lower cereal intake, resulting in reduced consumption of dietary fiber, while concurrently exhibiting higher consumption of animal-based foods and dairy, leading to an increased intake of cholesterol, red meat, processed meat, and dairy products. For instance, the Wellbama Program at Southeastern University analyzed generational differences in dietary fiber and cholesterol intake between Baby Boomers (born 1946–1964) and Generation X (born 1965–1979). The findings demonstrated significant generational differences in both fiber and cholesterol intake (*p* < 0.05). Specifically, Baby Boomers (12.3%) reported consuming 5–6 servings of fiber per day, compared to Generation X (8.2%). Moreover, nearly 80% of Baby Boomers reported consuming less than 2 servings of cholesterol per day, compared to less than 70% of Gen X [24]. Another study delved into the role of generational status on the dietary behaviors of Asian Americans and noted intriguing insights. Among all Asian Americans, 2nd-Gen participants reported higher intakes of red and processed meats (+0.044 servings/day; +0.029 servings/day), while 3rd-Gen participants exhibited higher processed meat intakes (+0.061 servings/day) when compared with 1st-Gen participants. In the context of East Asian adults, 2nd-Gen participants reported higher dairy and whole grain intake (+0.075 cups/day; +0.073 ounces/day) [10]. Nonetheless, it is worth noting that not all the results of foreign studies on generational differences in food consumption aligned with our findings. For example, an exploration of generational dietary differences among ethnic minorities in the UK revealed that only 2nd-Gen Indians consumed significantly fewer fruits and vegetables than the 1st-Gen, and no generational differences were observed in the diets of most other ethnic minorities [25].

Furthermore, research conducted in other countries has investigated generational differences in dietary patterns. Among the Pacific people of New Zealand, three distinct dietary patterns were identified: the “healthy diet,” the “processed diet,” and the “mixed diet.” The older generation predominantly adhered to a “healthy diet,” while the younger generation displayed a higher likelihood of consuming a “processed diet”. [11]. Similarly, generational differences in dietary patterns were observed among Brazilian adults from 1934 to 1975. Findings revealed a higher frequency of consumption patterns characterized by low-nutrient diets among the younger Generation X, with less energy intake (KJ/Kcal) coming from fruits (1502.5/359.1) and vegetables (311.3/74.4) compared to the Traditionalists (born 1925–1942) and Baby Boomers (born 1943–4960). Intriguingly, even with a higher total energy intake, the younger generation’s consumption of high-nutrient, low-energy-density foods lagged behind that of the older generation. This disparity may potentially result in the younger generation bearing a greater burden of chronic diseases as they age [9].

In Dalmatia, Croatia, potential triggers of generational shifts in the Mediterranean diet may stem from urbanization, population growth, and the progressive globalization of food markets [26]. Asian American dietary behaviors have been shaped by a multitude of social, cultural, and structural influencers within the context of the development of Western and Asian diets. This complex interplay has led to independent influences of generational status on eating habits [10]. The dietary patterns observed among different generations within Pacific peoples may be associated with sociocultural aspects related to eating habits [11]. Notably, generational differences in dietary patterns have been evident on both domestic and international levels, primarily driven by economic and cultural globalization, which has brought about changes in food consumption habits among younger generations.

This study possessed several strengths that should be highlighted. It offered an extensive examination of the dietary behaviors across various generations of Chinese residents, including the consumption of foods such as cereals, animal-based foods, vegetables, fruits, and dairy, as well as energy intake and the contribution of macronutrients to energy. In addition, this study carefully considered the effect of age on dietary behaviors when analyzing the generational differences and conducted a comparative analysis of diets among different generations within six age groups. However, like any study, certain limitations should be considered when interpreting the results. Firstly, the presence of non-Gen 10 individuals in the age group of 70 years and older could potentially introduce bias into the analysis of generational differences. Secondly, this study did not analyze the causes of generational differences in food consumption among Chinese residents, leaving room for further exploration in future research.

## 5. Conclusions

In summary, evident generational differences in food consumption were observed among different generations of adult residents in China. Younger generations displayed lower daily cereal and vegetable intake, energy intake, and the contribution of carbohydrates to energy compared to older generations (*p* < 0.0001). In contrast, the younger generation had higher daily animal-based food intake, average fruit and dairy intake, the contribution of protein to energy, and the contribution of fat to energy than the older generation. However, it is noteworthy that while the intake of fruit and dairy increased in the younger generation, their intake still fell short of the recommended nutrient intake levels. Unfortunately, the contribution of fat to energy tended to increase with age across all generations of residents. The dietary behavior of the younger generation not only reflected a portion of China’s current food consumption, but also provided a glimpse into its dietary future. Therefore, if these dietary trends persist, they could significantly impact the overall health of the Chinese population. Moving forward, it is imperative for relevant institutions and stakeholders to collaboratively develop public health interventions tailored to the youngest generation. Additionally, these entities should conduct further research to understand dietary patterns across the entire lifespan. These efforts will enable them to effectively advocate for healthy eating practices and the prevention of diet-related chronic diseases.

## Figures and Tables

**Table 1:** Birth generations corresponding to each age group in different survey years.

	1991	2000	2009	2018
20–29	Gen * 60	Gen 70	Gen 80	Gen 90
30–39	Gen 50	Gen 60	Gen 70	Gen 80
40–49	Gen 40	Gen 50	Gen 60	Gen 70
50–59	Gen 30	Gen 40	Gen 50	Gen 60
60–69	Gen 20	Gen 30	Gen 40	Gen 50
≧70	Gen 10	Gen 20	Gen 30	Gen 40

* Gen, generation.

**Table 2:** Basic characteristics of participants (N = 40,704).

	1991		2000		2009		2018	
	Men (n = 3835)	Women (n = 3914)	Men (n = 4667)	Women (n = 4657)	Men (n = 4580)	Women (n = 4790)	Men (n = 6673)	Women (n = 7588)
Age, (year)	42.7 ± 14.9	43.3 ± 15.4	45.7 ± 14.9	47.2 ± 14.8	50.4 ± 15.1	51.4 ± 15.0	54.4 ± 14.6	54.4 ± 14.7
Age groups, n (%)								
20–29	950 (24.8)	916 (23.4)	770 (16.5)	567 (12.2)	454 (9.9)	387 (8.1)	351 (5.3)	392 (5.2)
30–39	952 (24.8)	989 (25.3)	1061 (22.7)	1080 (23.2)	766 (16.7)	754 (15.8)	864 (12.9)	1022 (13.5)
40–49	780 (20.4)	787 (20.1)	1123 (24.1)	1214 (26.1)	1031 (22.5)	1123 (23.4)	1372 (20.6)	1542 (20.3)
50–59	550 (14.3)	581 (14.8)	844 (18.1)	813 (17.4)	1106 (24.2)	1165 (24.3)	1520 (22.8)	1719 (22.7)
60–69	414 (10.8)	395 (10.1)	537 (11.5)	605 (13.0)	725 (15.8)	761 (15.9)	1591 (23.8)	1810 (23.8)
≧70	189 (4.9)	246 (6.3)	332 (7.1)	378 (8.1)	498 (10.9)	600 (12.5)	975 (14.6)	1103 (14.5)
Education, n (%)								
Primary school or below	1887 (49.2)	2569 (65.6)	1715 (36.8)	2498 (53.6)	1510(33.0)	2459 (51.3)	1534 (23.0)	2764 (36.4)
Junior High School	1185 (30.9)	817 (20.9)	1598 (34.2)	1127 (24.2)	1756 (38.3)	1337 (27.9)	2295 (34.4)	2221 (29.3)
Senior high school or above	736 (19.2)	494 (12.6)	1223 (26.2)	835 (17.9)	1302 (28.4)	989 (20.7)	2832 (42.4)	2586 (34.1)
Place of residence, n (%)								
Rural	2484 (64.8)	2497 (63.8)	3214 (68.9)	3099 (66.5)	3164 (69.1)	3251 (67.9)	4018 (60.2)	4511 (59.5)
Urban	1351 (35.2)	1417 (36.2)	1453 (31.1)	1558 (33.5)	1416 (30.9)	1539 (32.1)	2655 (39.8)	3077 (40.6)
Working situation, n (%)								
Yes	3284 (85.6)	2962 (75.7)	3585 (76.8)	2995 (64.3)	3126 (68.3)	2426 (50.7)	3482 (52.2)	2841 (37.4)
NO	542 (14.1)	938 (24.0)	1033 (22.1)	1628 (35.0)	1453 (31.7)	2364 (49.4)	3180 (47.7)	4732 (62.4)
Individual annual income, n (%)								
Low	1288 (33.6)	1292 (33.0)	1538 (33.0)	1516 (32.6)	1465 (32.0)	1621 (33.8)	1875 (28.1)	2210 (29.1)
Medium	1243 (32.4)	1337 (34.2)	1542 (33.0)	1514 (32.5)	1524 (33.3)	1565 (32.7)	1935 (29.0)	2150 (28.3)
High	1300 (33.9)	1280 (32.7)	1511 (32.4)	1544 (33.2)	1545 (33.7)	1540 (32.2)	1947 (29.2)	2139 (28.2)

Values are mean ± SD or (M, IQR) or n (%).

## Data Availability

The datasets generated and analyzed during the current study are available from the corresponding author (H.W.) upon reasonable request.
